# OSCAR: A Modular Open-Source Robotic Platform for Biological Laboratories

**DOI:** 10.1021/acssynbio.5c00733

**Published:** 2026-03-09

**Authors:** David Pivin, Antoine Champie, Mirco Plante, François Ferland, François Michaud, Sébastien Rodrigue

**Affiliations:** 1 Département de génie électrique et génie informatique, Université de Sherbrooke, Sherbrooke, Quebec J1K 2R1, Canada; 2 Département de biologie, Université de Sherbrooke, Sherbrooke, Quebec J1K 2R1, Canada; 3 Département de biologie, 113603 Collège Montmorency, Laval, Québec H7N 5H9, Canada

**Keywords:** collaborative robotics, laboratory automation, task planning, open-source platform, molecular biology, DNA cloning

## Abstract

Biological research often involves complex, repetitive, and high-throughput manipulations that are well-suited to automation. However, current robotic systems generally excel only at narrowly defined tasks or standardized workflows and remain expensive, inflexible, and dependent on proprietary modules or reagents. To address these limitations, we developed the Open-Source Collaborative Automation & Robotics (OSCAR) platform, a flexible and low-cost system designed to perform common laboratory manipulations using standard, human-operated equipment. OSCAR incorporates open-source software and modular hardware to maximize accessibility and affordability. The platform features a robotic arm equipped with a dual-function end-effector: a pipetting module for precise liquid handling and a vision-enabled gripper for manipulating laboratory tools. To demonstrate the platform’s versatility, we implemented a representative plasmid assembly workflow, from PCR amplification and enzymatic assembly to transformation, plating, colony picking, PCR screening, and validation by agarose gel electrophoresis. By making this system open-source and compatible with widely used consumables and equipment, we aim to democratize access to automation and broaden its adoption across diverse research environments.

## Graphical abstract

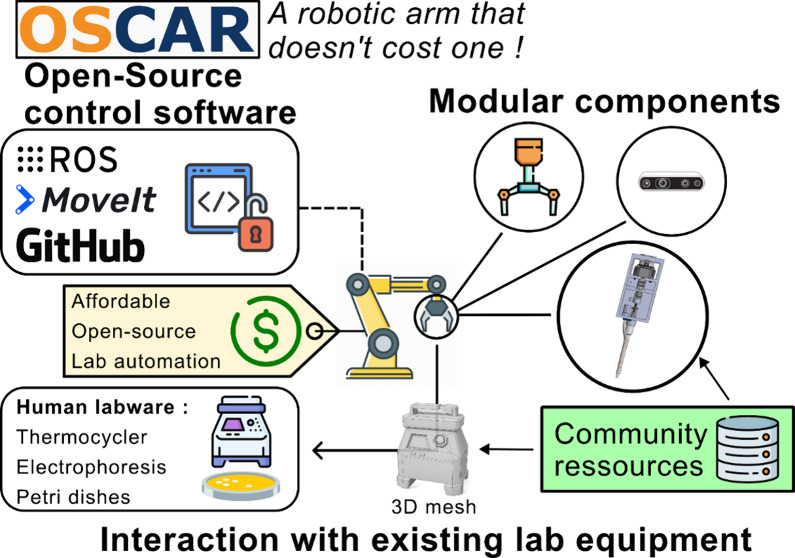

## Introduction

Robotic platforms are increasingly adopted across life-science disciplines, where they can improve reproducibility, accelerate experimentation, and enhance laboratory safety. A broad range of commercial systems now supports laboratory automation, spanning everything from compact liquid-handling instruments to large-scale installations integrating multiple coordinated robots. These offerings reflect the growing breadth and maturity of laboratory automation technologies. Nevertheless, most platforms remain tailored to repetitive, well-defined tasks, with substantial flexibility typically reserved for high-end systems. As a consequence, despite strong uptake in industrial and clinical settings, adoption in academic laboratories has lagged. Beyond the substantial upfront investment required to acquire an automation platform, commercial systems often rely on proprietary reagents and service contracts, which can rival the cost of the equipment itself.^1^ This steep financial commitment, combined with the high diversity protocols and the short-term nature of grant-based funding in academic research, can render specialized platforms obsolete or unsustainable within just a few years. As a result, many academic laboratories remain reluctant to adopt automation, continuing to rely heavily on manual labor often performed by trainees.^1^

The shift toward human-centric automation, exemplified by the Industry 5.0 framework,^2^ is advancing robotics toward more collaborative designs instead of rigid systems that often execute narrowly predefined tasks constrained by fixed commercial modules. This new paradigm increasingly emphasizes flexibility, adaptability, and ease of interaction. These ideas are now influencing laboratory automation, motivating the development of robotic systems intended to assist researchers across a broader range of experimental workflows. Despite recent advances and the emergence of several capable platforms, achieving general-purpose robotic manipulation for laboratory tasks remains an open challenge. For instance, Ding et al.^3^ reported a do-it-yourself integration strategy that incorporates a microplate imager into an existing mammalian cell line development automation platform. Their approach uses the open-source AutoIt scripting language to coordinate imaging within a workflow already equipped with a 4-axis robotic arm and an automated incubator, enabling automated scan-profile selection and both automated and remote error handling. By focusing on instrument integration rather than broad manipulation capabilities, the system effectively automates a specific but essential step in the cell-line development process, while remaining constrained by the kinematic and functional scope of the underlying platform. A recent example of a six–axis–based system is the modular platform developed by Hamm et al.,^3^ which automates multistep biological workflows with a focus on cell culture and gene transfection experiments. The platform supports automated tool changes and executes tasks through a trajectory-centric strategy in which motion paths are predefined and validated. This method is effective in stable and well-characterized layouts and reflects a design choice that prioritizes reliability and repeatability. However, it typically requires device- or layout-specific retuning when labware geometry or placement changes, sometimes necessitating full replanning. The liquid-handling demonstrations use a two-finger gripper to manipulate different pipettes via position control, with slightly more variability than a skilled human operator. Another interesting strategy is demonstrated by ORGANA,^4^ a robotic assistant designed for automated chemistry experimentation. The platform integrates high-level task and motion planning with a library of predefined laboratory skills, and leverages large language models to translate natural language instructions into executable procedures, substantially reducing the programming burden for nonexperts. Although ORGANA uses advanced motion-planning algorithms such as PRM* to generate collision-free trajectories, these sampling-based approaches are inherently nondeterministic, leading to variable planning times and trajectory quality. In addition, liquid handling is largely delegated to an integrated syringe-pump system, which is poorly suited to many biological workflows that require fine positional control and versatile pipetting. Together, these features illustrate meaningful progress toward more accessible laboratory robotics while underscoring the persistent difficulty of achieving general-purpose manipulation in complex research environments.

As a step toward broader adoption of automation in life science laboratories, we introduce the Open-Source Collaborative Automotion & Robotics (OSCAR) platform, featuring a 6-degree-of-freedom robotic arm equipped with a pipetting tool, a force-torque sensor, and a vision-enabled versatile gripper to interact with standard laboratory equipment. The system represents labware using simple semantic labels and object-specific interaction rules, allowing tasks to be defined relative to the objects themselves rather than fixed robot coordinates. For operations that require precise physical contacts, the platform can switch from position control to force/compliance control, improving robustness to geometric tolerances and enabling reliable low-volume pipetting (e.g., ∼ 1 μL) at the bottom of wells. We demonstrate OSCAR’s capabilities by performing a generic plasmid cloning protocol, which included PCR amplification, Gibson assembly, transformation into *Escherichia coli* competent cells, plating, colony picking, PCR validation, and agarose gel electrophoresis. In our experiments, each protocol could be planned in under 5 s and executed autonomously. The same protocols were successfully simulated on two additional robotic platforms without rewriting the task logic. This simple workflow showcases a set of basic actions that could be reassembled into a wide variety of protocols for various research objectives.

## Results

### Description of the Platform Components

The OSCAR platform (Figure 1A) consists of a generic-purpose six degrees of freedom UR3 robotic arm from Universal Robots, selected for its accessibility (<$20,000 USD), high repeatability (≤0.1 mm), and extensive compatibility with third-party accessories. The robotic arm is mounted on a 1 m x 1 m stainless steel optical table with mounting holes, forming a 1-in. grid. A force torque sensor is installed at the end of the robotic arm, onto which a dual-function effector is mounted. This effector integrates a custom-built, electronically controlled micropipette and a versatile gripper.

**Figure 1:**
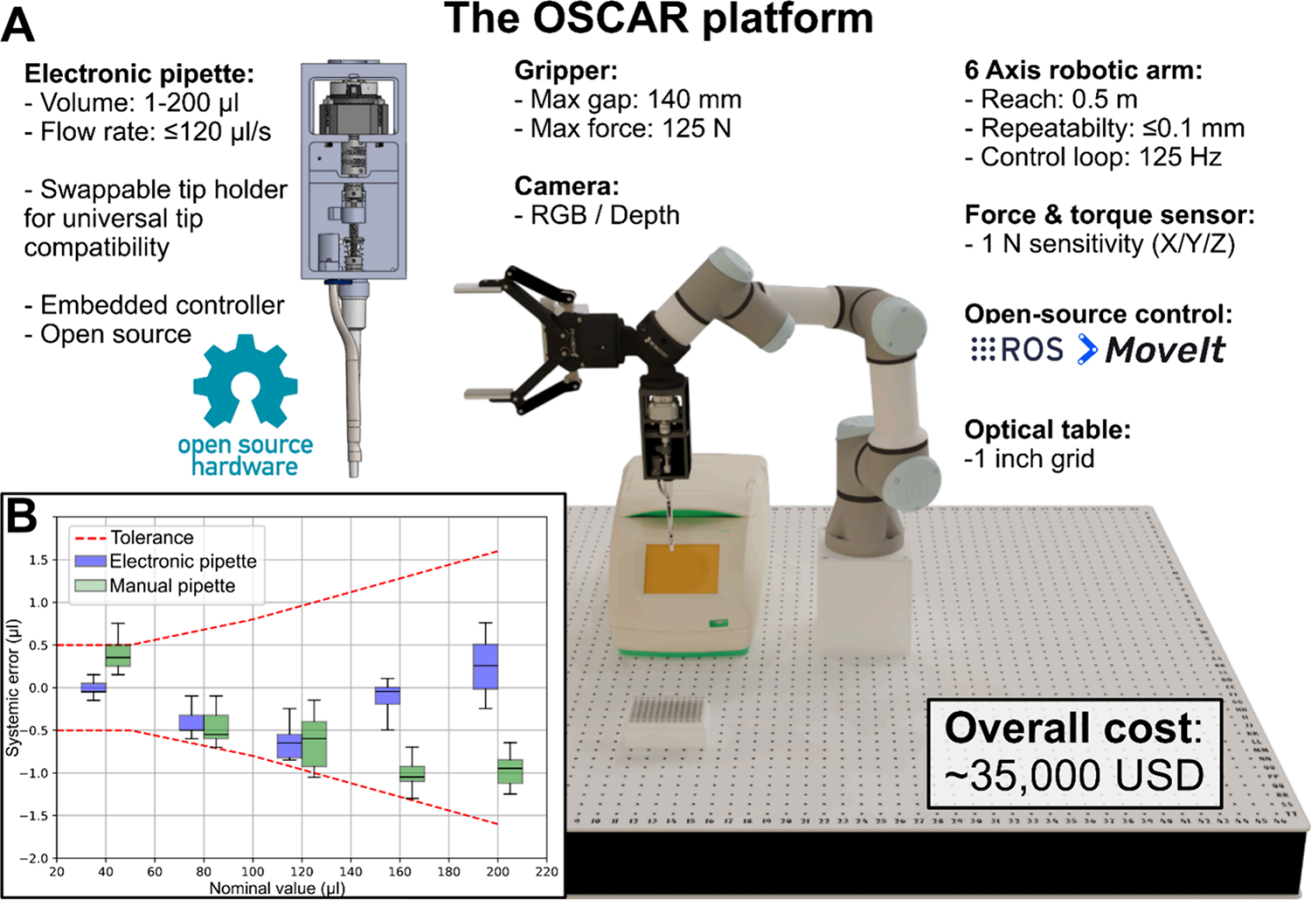
Overview of the OSCAR robotic platform. A) 3D rendering illustrating the si*x*-axis robotic arm in the configuration used in this manuscript, including a dual-function effector composed of a versatile gripper and a custom-made electronic pipet. The thermocycler and tip-carrying box are displayed for scale. The total cost includes all displayed elements, except for the thermocycler. B) Pipetting accuracy comparison of the performance of the custom-built electronic pipet (blue boxes) or a manual pipet (green boxes) against the manufacturer’s specified tolerances (red dashed lines). Box and whiskers represent quartiles, and *N* = 10 for each measurement.

Although we aimed to reuse existing equipment and labware whenever possible, direct manipulation of a standard manual pipet by the OSCAR proved impractical. Instead, we elected to design an open-source micropipette that integrates the mechanism of a Gilson manual micropipette, with the piston controlled by a microstepper motor housed in a 3D-printed frame. This pipet head can accurately dispense volumes ranging from 1 to 200 μL (Figure 1B and Figure S1) using standard 200 μL laboratory tips taken from a regular tip box held by a 3D-printed adapter. The pipet shaft can be easily swapped with another Gilson brand or 3D-printed alternative to accommodate any tip brand. Using this custom system, we achieved precise Cartesian positioning of the pipet tip, enabling accurate operations such as tip insertion, which requires a precision of ± 0.5 mm, and picking colonies as small as ∼ 1 mm in diameter.

On the opposite side of the dual-function effector is a versatile gripper, capable of handling objects measuring up to 140 mm and weighing up to 2.5 kg. The gripper is primarily used for moving consumables, plates, opening Petri dishes, and interacting with other lab equipment. Its functionality is expanded by grabbable tools such as a stylus designed to mimic a human finger, facilitating interaction with buttons and touch-sensitive laboratory devices, as well as a customized cell spreader for distributing liquid across agar surfaces. Adjacent to the gripper, the system features an Intel RealSense D415 vision camera, which enables different functions such as colony picking, agarose gel inspection, and input validation during PCR machine programming using AI-assisted image processing.

### Motion Planning and Control Architecture

We developed a software platform that generates robot behaviors from digital models of the labware and a calibrated map of the workspace, rather than from device-specific waypoint recording or manual teaching. This model-based approach provides far more flexibility, since actions are described in terms of objects and their relationships rather than being tied to a single robot or bench layout. The following paragraphs offer a user-oriented description of this planning process, with additional details and extensive justification for the design choices available in the Supporting Information.

At its core, the OSCAR platform bridges biology-native actions (e.g., “open lid”, “pipette”, “spread”, “press button”) to robotic execution using reusable nodes that can be assembled into full experimental workflows. The platform is organized as a layered system that handles all aspects of planning, from protocol specification and semantic object modeling to task coordination, motion generation, and low-level execution, thus allowing the same high-level protocol logic to be deployed across different workspace layouts and robot manipulators.

The platform operates using the Robot Operating System (ROS),^5^ an open-source software development framework that enables communication between devices or modules using standardized messaging protocols. ROS is widely adopted for building advanced robotic applications and is currently used to control a large fraction of robotic systems worldwide.^6^ This unified communication system enables seamless integration of heterogeneous devices, such as force–torque sensors, and keeps high-level logic independent of the implemented hardware. As a result, the same software components and interfaces can be used interchangeably in simulation and on real hardware, facilitating rapid development, debugging, and deployment.

For motion generation, the platform uses ROS tools such as MoveIt for kinematics, collision checking, and motion planning, and the MoveIt Task Constructor (MTC) to organize complex manipulations into modular steps. Although the system operates within ROS, it is not limited to ROS-native tools. External libraries, including our newly developed Domain-Specific Language (DSL) and the Semantic Object Description Format (SODF) library, can be integrated seamlessly. Figure 2 summarizes the high-level software interactions and the corresponding hardware connections.

**Figure 2:**
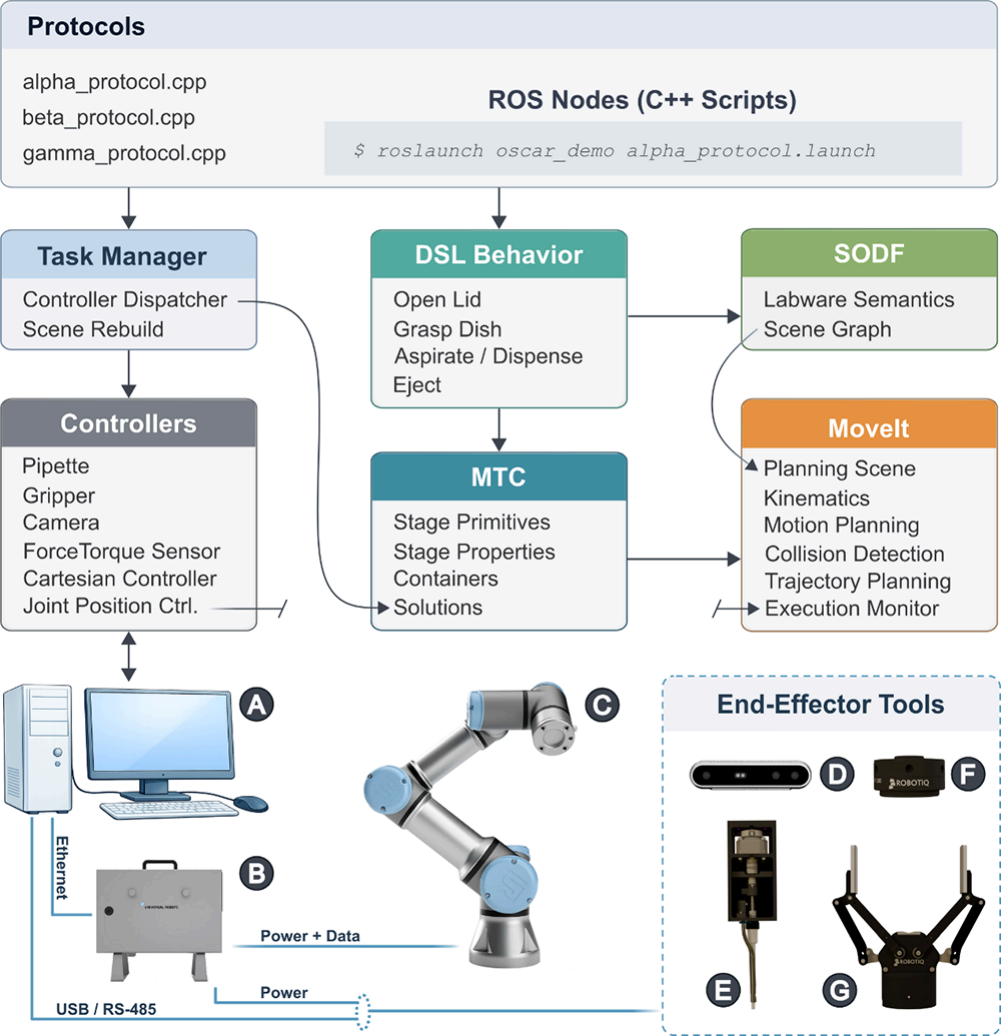
OSCAR high-level architecture for software and hardware components. Laboratory protocols are implemented as C++ nodes and launched as executable workflows that invoke a task manager to dispatch actions to the appropriate controllers. High-level manipulation behaviors are expressed using the DSL, which uses the composition of the workspace (provided in SODF) to plan behaviors. These behaviors are compiled into motion plans using MTC, while MoveIt provides kinematics, collision checking, and trajectory planning. Execution is carried out through a set of controllers and drivers. The lower part of the figure illustrates the physical system, including the control PC, robot controller, robotic arm, and end-effector tools (camera, pipet, force-torque sensor, gripper), connected via Ethernet, USB/RS-485, and power lines.

Protocols are written using a newly developed Domain-Specific Language^7,8^ (DSL) tailored to robotic laboratory automation, in which workflows are expressed as sequences of well-defined domain “actions” (e.g., open lid, grasp, insert tip, aspirate, dispense/mix, spread, eject tip). Each “action” includes easily accessible parameters such as target objects, volumes, speeds, and force settings. The DSL provides protocol authors with an accessible high-level interface, implemented to facilitate reuse and protocol tweaking while keeping low-level protocol logic accessible for more advanced users.

An overview of the “actions” and the relationships between parameters, semantic object models, planners, and controllers is provided in Figure 3 for the simple case of aspirating a liquid. In this simple example, two action nodes, InsertTip and AspirateLiquid, are used to collect 5 μL of liquid from well A1 of a 96-well plate using a new pipet tip. Defaults parameters allow protocols to be specified without expert robotic knowledge, but all high-level and low-level parameters may be adjusted at will. The *InsertTip* action uses a Cartesian path, combined with a force controller, to apply a 25 N insertion force on the pipet tip at the target location. The *AspirateLiquid* action operates in automatic mode, using position control to lower the tip in the well until a height threshold is reached, at which point force control is enabled for the rest of the descent. In this example, aspiration is performed 5 mm below the liquid surface with a 7° tool inclination. The DSL actions encode insertion affordances (the feasible approach orientations permitted by the geometry of the pipet and container), allowing the task planner to explore multiple insertion angles if an initial attempt fails. The angled aspirating motion is shown solely to demonstrate the framework’s ability to orient the tool according to user-defined parameters. By default, aspiration is performed vertically for accuracy, but dispensing can be executed at an angle, for example to direct liquid against the wall of a tube.

**Figure 3:**
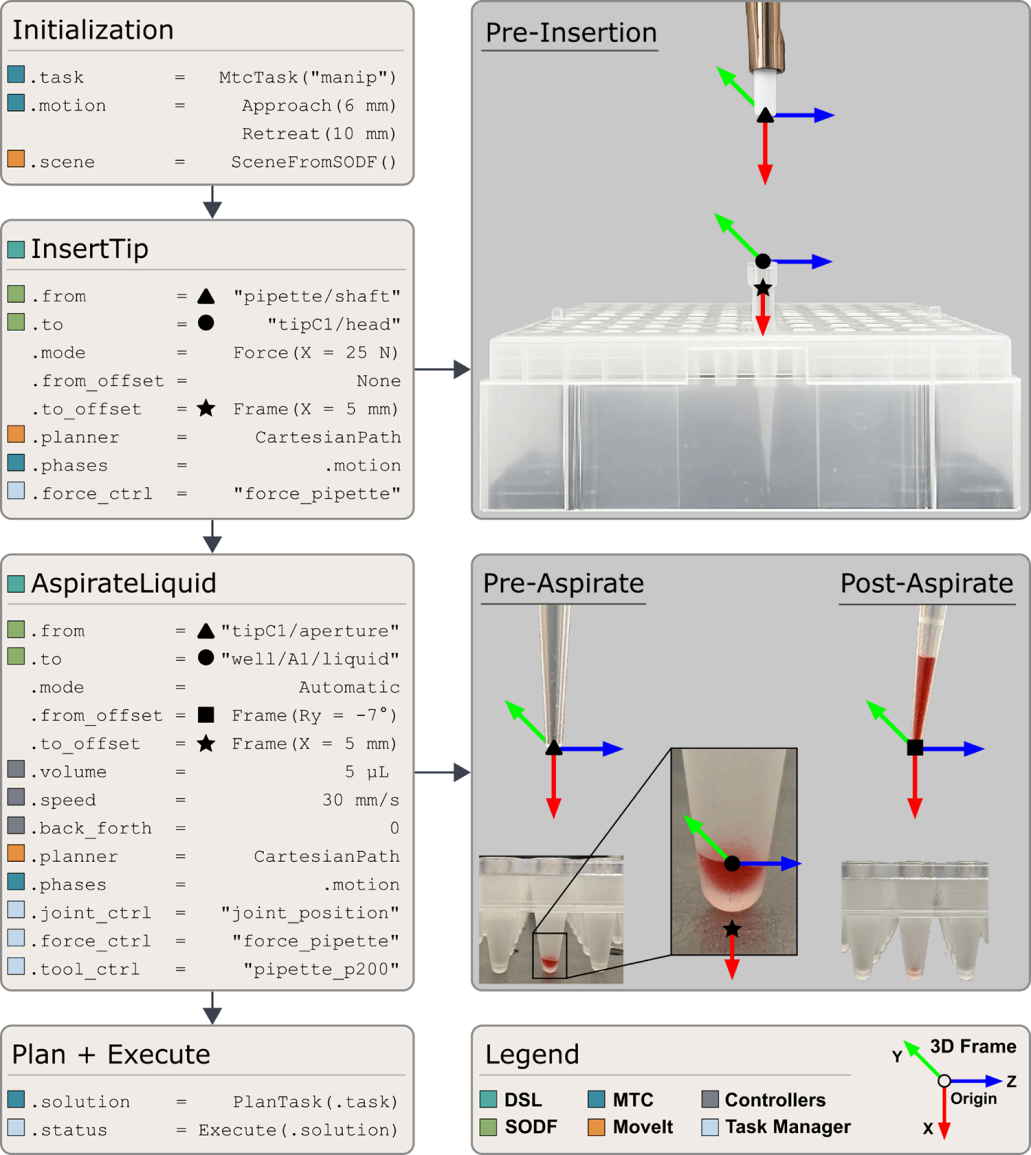
Representative laboratory manipulation encoded with DSL action nodes. The left column shows two high-level DSL action nodes, *InsertTip* and *AspirateLiquid* and their associated parameters. Parameter categories indicated by colored boxes corresponding to the system architecture they interact with. The right column illustrates the SODF source and target frames that the task planner attempts to align, each optionally supplemented with a frame offset.

To enable manipulations requiring precise and/or delicate physical interactions with labware, our approach relies on a precise description of each piece of labware. For this purpose, we developed the Semantic Object Description Format (SODF), a library that represents laboratory equipment through both geometric models and defined interaction points. Creating the SODF equivalent of lab equipment requires recording its key physical features, including its 3D shape, button positions, center of mass, and interaction forces. Following this, each object is precisely anchored onto the table using precision shoulder screws and 3D-printed supports. This teaching-agnostic approach makes the robot more versatile: once the physical objects have been precisely characterized, OSCAR can automatically adapt to their changing positions on the work surface. SODF also encodes container geometry and liquid-surface information, allowing the robot to estimate liquid height and use it for precise low-volume manipulation; a capability largely missing from existing open-source robotic planning systems. This characterization process can be time-consuming if CAD models cannot be obtained from the manufacturer. However, once created, the resulting SODF files are portable and can be shared between users and used across various implementations of OSCAR regardless of the specific robot configuration.

Task coordination is handled by the MTC library,^9^ which divides each protocol into small planning steps, optimizes them separately, verifies compatibility between those independent planning and arranges them into final executable workflows (Figure S2A). Planning of each step uses the labware’s shape and interaction points derived from the SODF object models. For instance, pressing a thermocycler button with a stylus is specified by defining a source frame (the stylus tip) and a target frame (the button), such as ″stylus/insert/tip″ and ″thermocycler/button/incubate″, which are passed as parameters to DSL actions like PushButton. The implied geometric relationships are automatically resolved during planning, so the user only needs to specify the intended interaction. MTC is well suited for long, multistep protocols, as it can detect failures early, supports fallback strategies, and selects the best valid motion among alternatives (Figure S2B). This entire planning process can be visualized using RViz, providing insight into task progression. Task execution is then handled by a dedicated Task Manager, which aggregates all valid solution candidates generated by MTC and selects the solution with the lowest cumulative joint displacement, favoring efficient motions (Figure S2C).

Motion planning is performed using a dual-mode strategy (Figure S3) that combines MTC-generated trajectories for noncontact motions with reactive force-based control for contact interactions. The same robot controller is used in both modes: it receives precomputed trajectories during noncontact motion, and dynamically generated trajectories based on real-time force sensing during contact. Those motion-planning steps are primarily performed using the MoveIt library,^10^ which already contains a mature suite of trajectory generators and collision-checking mechanisms. An example of this dual-mode strategy is shown in Figure S4, where an aspiration action is broken into a standardized sequence of motions. These standardized steps enable robust and fast planning, facilitate collision avoidance near contact zones, and support real-time substitution with compliant reactive force-based control when required. Overall, this modular architecture makes the system ideal for robustly executing biological protocols by efficiently chaining together a wide variety of generic operations, including (Figure 4):

1. Accurate pipetting of volumes ranging from 1 μL to 200 μL.
2. Tip insertion and ejection.
3. Manipulation of labware with the gripper, such as 96-well plates, plate covers, and Petri dishes, as well as the operation of the thermocycler’s door.
4. Machine interaction using a stylus attachment, including touchscreen inputs for thermocycler programming or interacting with buttons on other machines, such as electroporators and gel electrophoresis power supplies.
  Spreading bacteria on Petri dishes with a spreader attachment.
5. Using the integrated camera to capture images of agarose gels postelectrophoresis, verify machine inputs, and locate colonies on Petri dishes.

**Figure 4:**
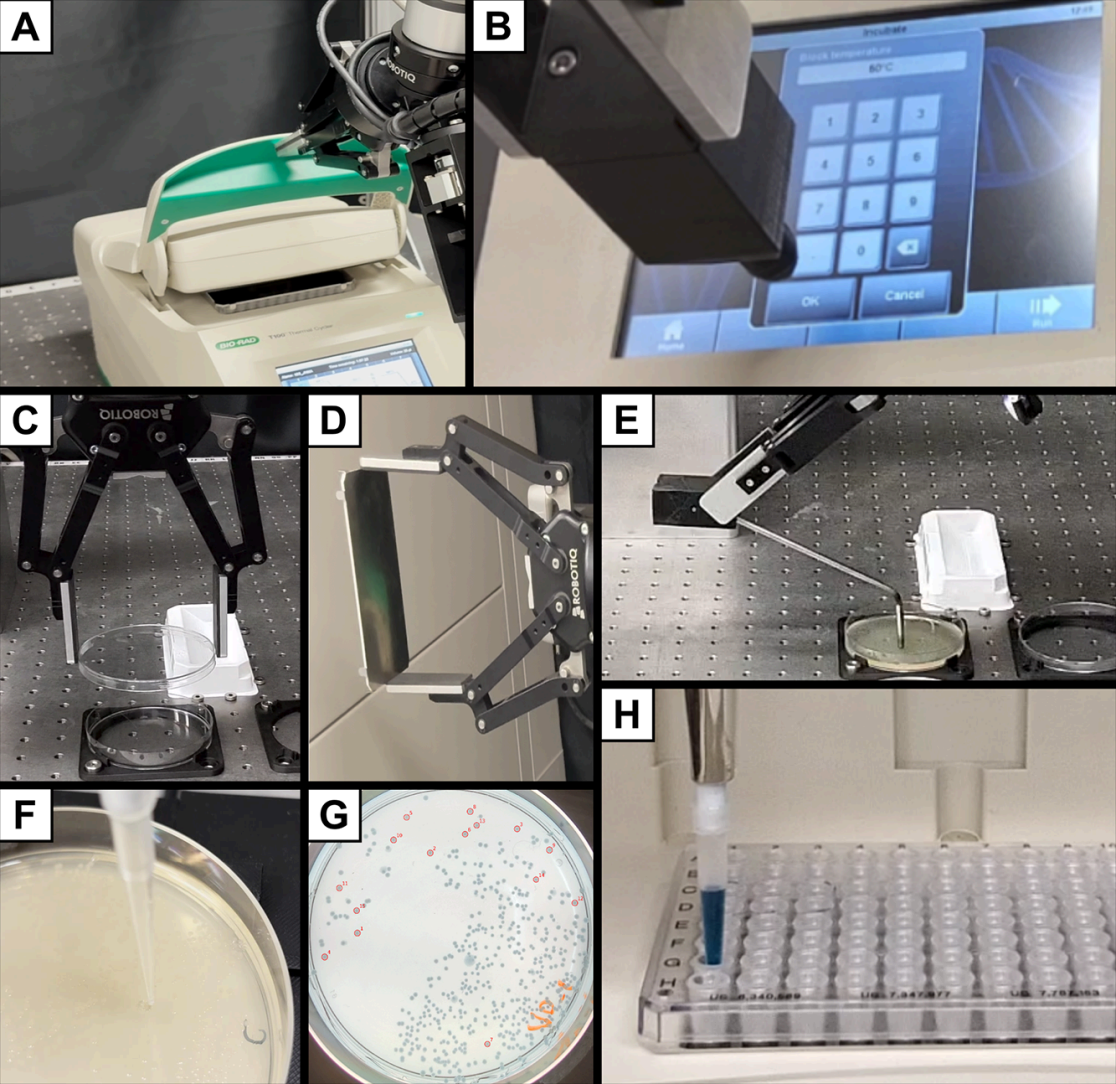
Laboratory manipulation tasks demonstrating the capabilities of OSCAR’s modular system architecture. Automated interaction with a thermocycler, including lid manipulation (A) and touchscreen programming using a stylus end-effector (B); manipulation of labware using a parallel gripper, such as grasping a Petri dish lid (C) and a reusable PCR cover (D); automated bacterial spreading using a dedicated spreader attachment (E); locating colonies on Petri dishes using the integrated camera (F); accurate liquid handling with pipetting volumes ranging from 1 μL to 200 μL, including tip insertion, aspiration, and dispensing into multiwell plates (G) and Petri dishes (H).

### Demonstration of OSCAR’s Biological Manipulation Capabilities

To demonstrate the capabilities of the OSCAR platform, we conducted a series of three successive protocols showcasing the assembly of a plasmid and its validation (Figure 5). This workflow was selected for its widespread use in molecular biology laboratories and to showcase the key capacities of the system: precise liquid handling, interaction with human-designed instruments such as PCR machines (including automated lid opening, closing, and programming), colony picking, or agarose gel loading. These steps can be rearranged to create a wide range of similar protocols, highlighting the platform’s flexibility.

**Figure 5:**
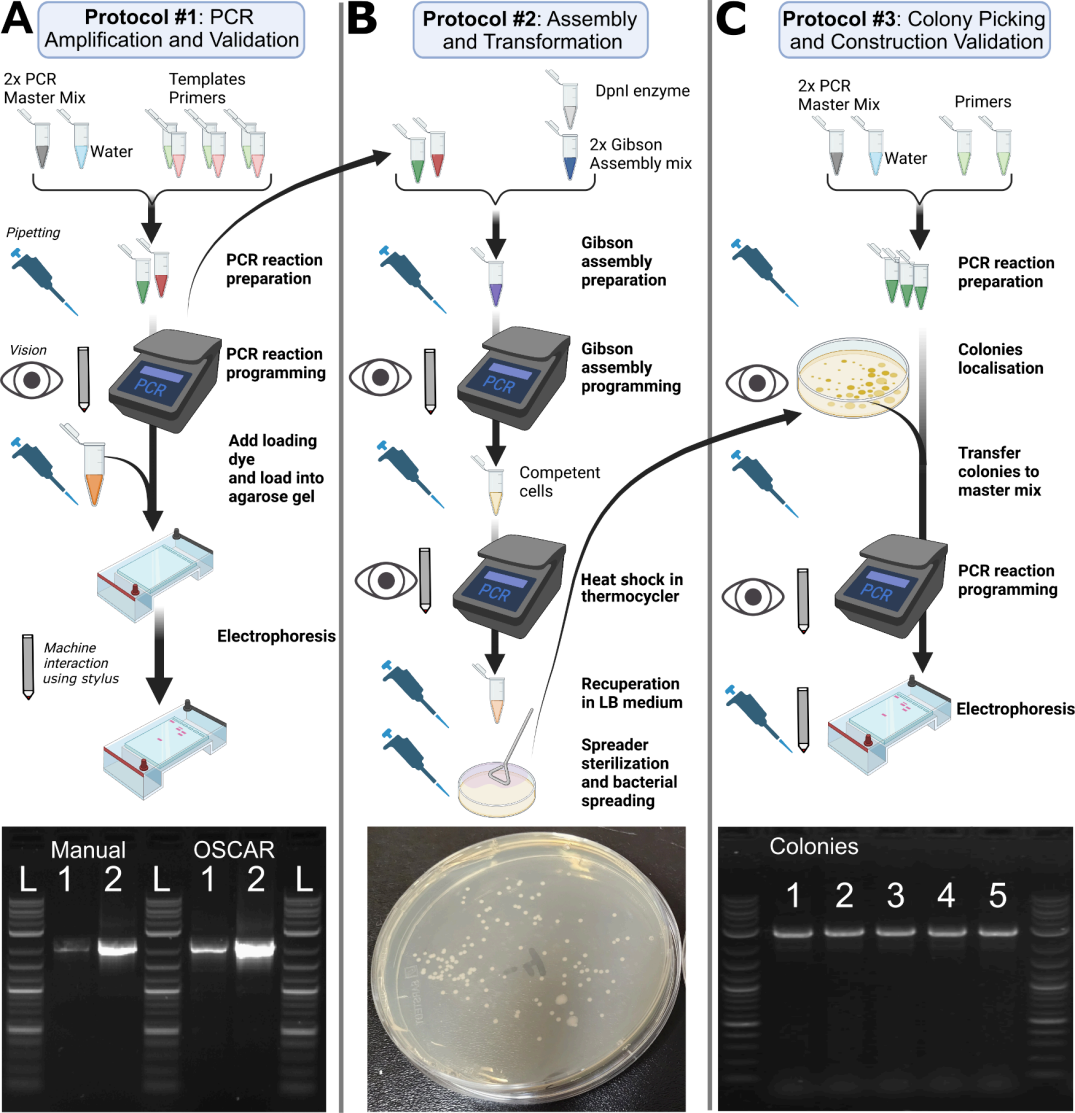
Overview of the three protocols performed by the OSCAR platform. A) PCR amplification and validation. Reaction preparation, thermocycler programming, agarose gel electrophoresis, and gel imaging steps are illustrated sequentially. Icons represent the modules used to interact with the environment: eye icon = vision, pipet icon = liquid handling, stylus icon = stylus. The resulting electrophoresis gel is shown below (L: DNA ladder; 1, 2 two fragments of the assembled plasmid, comparing manual vs robotic amplification). B) Gibson assembly and bacterial transformation. Steps include enzymatic assembly preparation, thermocycler-based incubation, transformation into competent cells, and plating. Transformation outcomes are displayed below. C) Colony picking and construct validation. Steps involve colony localization, PCR preparation from selected colonies, reaction programming, and subsequent electrophoresis for validation.

To highlight the robot’s ability to interact with standard lab equipment and demonstrate the low investment required to deploy the platform, we endeavored to interact with existing machines using computer vision and a stylus. While direct electronic control of accessory devices would have been easier to implement, this often requires specialized application programming interfaces (APIs) and costly proprietary equipment, if such options are even available. For all experiments, the workstation is prepared by arranging the necessary laboratory equipment in custom 3D-printed holders. The required reagents are dispensed into a 96-well plate, which is then placed in the thermocycler at 4 °C with a reusable cover. The required 1.5 mL tubes are manually opened and positioned in an aluminum block set within an ice-filled support.

Speed-up videos of the manipulations are available on an associated YouTube channel (link in Supporting Information), and detailed protocols are provided in File_S1. All custom 3D-printed models and CNC files referenced are also included on the associated GitHub: https://github.com/rodrigue-laboratory/pipette-tool-cad.

In Protocol #1 (Figure 5A), the robot amplifies two DNA fragments from template plasmids using PCR and verifies the fragment sizes via agarose gel electrophoresis. In the first step, the robot sets the thermocycler to 4 °C to keep reagents cold. It then uses the pipet to mix primers, template, and a 2X PCR mix to prepare the reactions. The robot automatically adjusts the aspirating and dispensing at target positions according to the precharacterized geometry of the well. To compensate for potential positional uncertainties, the control strategy transitions from “position control” to “force control” when the pipet approaches a user-defined distance from the bottom of the well. This ensures reliable aspiration regardless of the volume present in the tube, without risking damage or imprecise positioning.

OSCAR then uses its stylus to set up a PCR reaction and waits for it to be completed. Required user inputs are limited to specifying the current state of the PCR touchscreen interface and a sequence of target button presses. This high-level abstraction is made possible through prior characterization of the touchscreen layout and behavior. To enable reactive feedback, the system can optionally interface with the Google Vision API to assess the touchscreen state and confirm successful actuation of on-screen elements.

After the amplification finishes, the robot mixes 5 μL of each reaction with 5 μL of loading dye and uses the mixture to verify amplification quality using a horizontal DNA gel electrophoresis system. To facilitate this process, we designed and 3D-printed a comb with Y-shaped openings, allowing the robot to pipet without precise gel positioning in the tank while maintaining narrow, sharp bands on the gel. Additionally, we modified the electrophoresis tank lid by adding an aperture above the gel wells, allowing direct pipetting into the gel without removing the cover. The robot then grasps the stylus and uses it to activate the power supply. Attempts were made to automate the gel imaging process but were thwarted by excessive condensation in the electrophoresis tank, a problem which could be solved by using a different gel electrophoresis unit^11^ or designing a custom module. Following migration, the DNA fragments were thus manually validated in a UV transilluminator and purified using SPRI bead-based purification.

In Protocol #2 (Figure 5B), the robot assembles the previously amplified fragments into a single circular plasmid using the 2X commercially available NEBuilder HiFi DNA Assembly mix. The resulting DNA was then transformed into chemically competent *E. coli* and spread onto a selective Petri dish. To enable the robot to spread the transformed bacteria evenly on the Petri dish, we designed a custom spreader tool for the gripper. This tool consists of a standard steel bacterial spreader mounted on a free-axis inside a 3D-printed holder. With this setup, the only downward force exerted is the weight of the spreader itself, allowing for gentle and even spreading of the bacteria without damaging the fragile agar surface. To sterilize the spreader, it is dipped into a 70% ethanol basin for 30 s, shaken to remove excess liquid, and left to air-dry for a few seconds. Following bacterial spreading, the plates were manually placed in a 37 °C incubator overnight for colony growth.

In Protocol #3 (Figure 5C), the robot utilizes its vision system to locate colonies on the previously plated Petri dish and uses colony PCR to verify the correct assembly of the plasmid. A picture of the complete initial setup for this protocol is presented in Figure 6. To identify colonies on the Petri dish, the robot captures an image, which is then processed using a combination of filtering and watershed algorithms to detect isolated colonies (Figure S5 and Table S6). Once the colonies were located, five were picked using the pipet tool and resuspended in a previously prepared PCR master mix. After amplification, the resulting DNA is subjected to agarose gel electrophoresis as outlined in Protocol #1.

**Figure 6:**
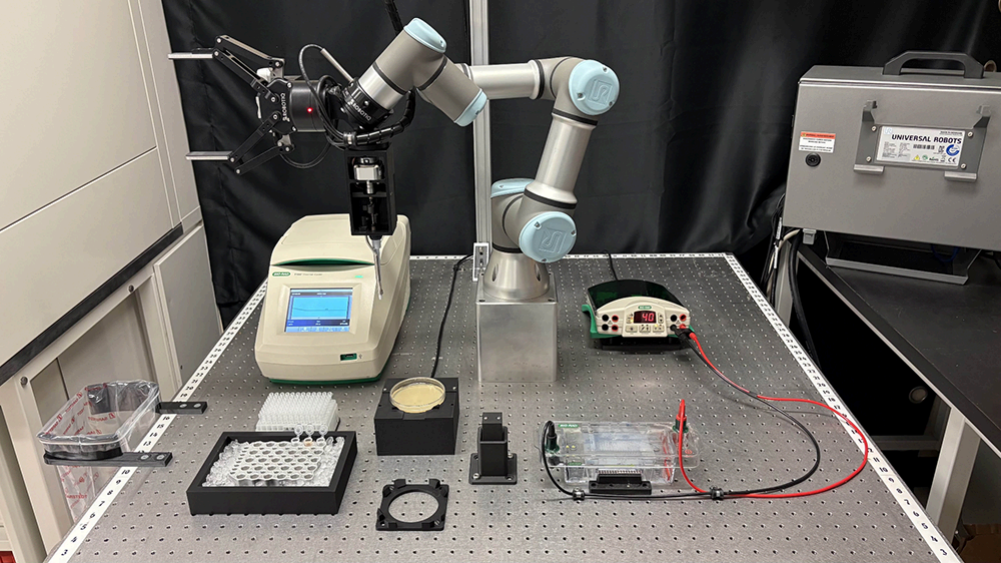
Experimental setup for Protocol #3. Setup includes from left to right: a 3D-printed tip disposal; a thermocycler in which a 96-well plate is stored covered by a reusable lid; a tip carrier; 1.5 mL tubes containing various reagents and kept on ice; Petri dish placed on a custom illuminating plate; Petri lid holder, stylus module; a horizontal agarose gel electrophoresis unit and its power supply. All elements are kept in place using 3D-printed supports compatible with the optical table.

### Portability of the OSCAR Platform across Robotic Systems

The OSCAR platform was designed with modularity and hardware flexibility in mind, allowing interaction with various laboratory instruments and interchangeable components. Even the UR3 arm, which was used for physical testing due to its low cost and accessibility, can be easily replaced by other arms with different capabilities, such as extended reach to support more equipment within a single protocol. To showcase the framework’s independence from specific robot models, we successfully replanned all three protocols using both a UR5 and a Franka Emika Panda arm, reusing the same task planning pipeline as with the UR3 (Figure 7). Overall, the planification time remain very short across all simulations with the different robotic arms, with an average of 5 s for complete protocols. Due to a more limited joint position constraint compared to the UR robots, the Panda robot cannot grasp the spreader at the original location used in Protocol #2. Resolving this issue required moving the location of this accessory further away from the base of the arm, a trivial adjustment thanks to the automated motion planning. We expect that the ability to interchangeably use a wide range of robotic arms, effectors, and laboratory equipment, depending on user preference, budget, or material availability, will broaden the potential applications for users with diverse needs.

**Figure 7:**
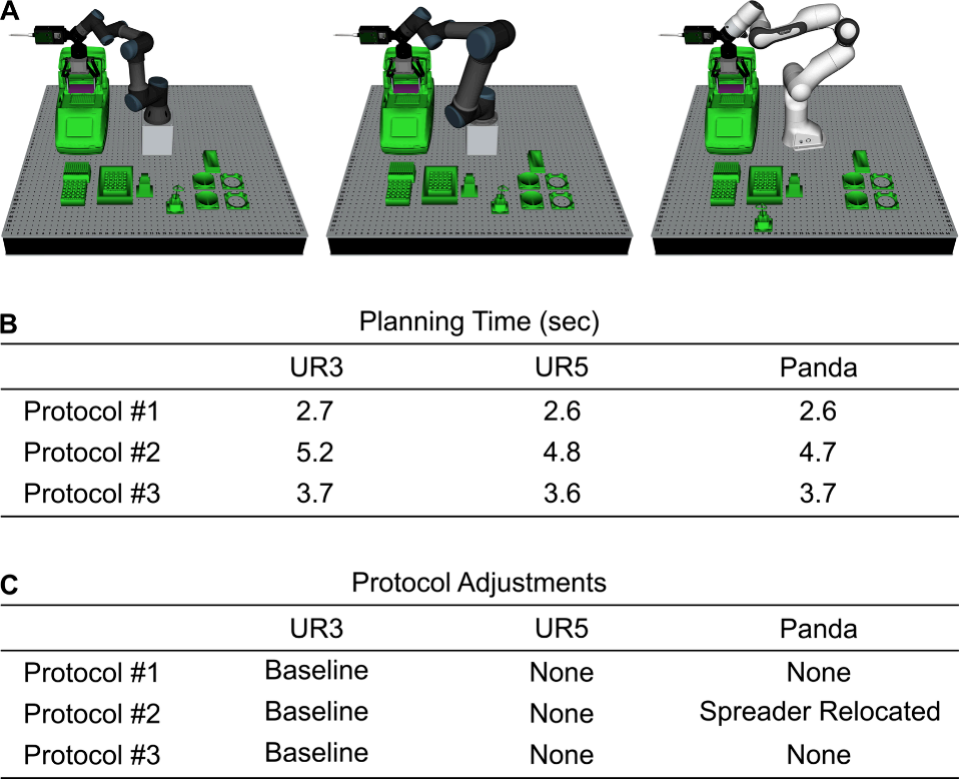
Planning time benchmark using alternative robotic arms. A) 3D visualizations of UR3, UR5, and Panda robot arms executing protocol #2. B) Planning time for each protocol using each arm, averaged over five repeated runs with standard deviations below 80 ms. Each protocol execution lasted approximately 10 min, performed on a Dell Inspiron 15–7577 equipped with 16 GB of DDR4–2400 RAM. C) Summary of protocol adjustments required for each robot relative to the UR3 baseline. Protocols 1 and 3 did not require any changes, while Protocol 2 required adjustments for the Panda arm, namely that the spreader had to be relocated further from the base due to limited joint-position constraints.

### Discussion

With the first iteration of the OSCAR platform, we present a flexible automation platform capable of performing various routine biological laboratory tasks, such as PCR amplification, gel electrophoresis, DNA assembly, and transformation into competent cells, using standard lab equipment and 3D-printed parts. OSCAR was built on a limited budget of approximately $35,000 USD, comparable to an entry level liquid-handler like an Opentron Flex robot equipped with a pipet and a gripper. While commercial systems typically rely on proprietary consumables for optimal performance, our solution was designed to work with standard laboratory consumables and equipment. Implementing an OSCAR platform costs a fraction of the price of a high-end lab automation system, which would be required to perform all those tasks and would typically reach hundreds of thousands of dollars, depending on the configuration. By leveraging existing laboratory equipment, the OSCAR platform is an accessible entry point for academic laboratories seeking to automate various manipulations. All components, including the codebase and peripheral tools such as automated pipettes, force-torque sensors, grippers, and cameras, are either open-source, easily 3D-printable, or commercially available from multiple suppliers. We anticipate that this ease of accessibility will lower the barrier to entry and facilitate adoption by other groups. While initially developed for molecular biology applications, OSCAR’s architecture is not restricted to this field, and its core features should readily be transferable to workflows in other experimental sciences. Community engagement would greatly enhance the project, as protocols can be shared between users, and any equipment characterized or open-source modules developed can be widely reused. The platform brings together several capabilities that enable flexible and reliable robotic lab automation. It combines position and force control, enabling the robot to handle contact interactions with greater consistency, including precise liquid handling at the microliter scale. Task execution is organized around reusable templates, enabling fast planning and straightforward application of the same protocol structure across different robotic platforms; notably, the same protocols can be deployed on three distinct robotic arms with minimal modification.

The OSCAR system also demonstrates long-term autonomy by planning and executing three complete protocols involving very diverse sets of manipulations for approximately 10 min of operation without human intervention, a level of continuous operation that, to our knowledge, remains challenging for laboratory manipulation systems.

With this solid foundation in place, we aim to further enhance the platform in several ways. The first area of improvement would be to enhance the user experience, specifically by simplifying the platform’s initial setup in a new environment. While a trained user can easily perform precise modeling of laboratory equipment, this step may pose challenges for inexperienced users. This process could be greatly streamlined by implementing a metrology-based pipeline for new object characterization (e.g., via 3D scanning). The current protocol planning strategy relies on user-provided equipment localization in the work area. While this approach is simple to use, it could be further accelerated by leveraging the robot’s vision capabilities to automatically detect and locate newly added equipment. Automatic detection of smaller biological labware could also be envisioned, but despite advancements in vision algorithms and object detection, it still presents significant challenges, as these objects are typically small, colorless, textureless, and may be stacked, making them among the most difficult categories for artificial vision systems to handle.^12^

The use of thermocyclers as general-purpose dry heater-cooler blocks demonstrates the platform’s versatility but may become limiting over time due to their large footprint. Development of open-source, electronically interfaceable alternatives to commonly used laboratory equipment, such as the custom electronic pipet we developed, could further reduce costs, expand the platform’s capabilities, and remove some of its current limitations. For example, the platform currently relies on preopened and prefilled tubes for the storage of high-volume (>200 μL) liquids. Although the robot’s precise pipetting minimizes dead volumes, this approach requires manual aliquoting and leads to some enzyme loss. This challenge could be addressed by characterizing and integrating a tube-sealing device or developing an automated tube opening/closing module like the one proposed by Jinno et al.^13^ Other modules that would significantly enhance the platform include a Peltier-based heating/cooling module and the integration of a magnetic bead purification system to enable SPRI-based DNA purification, which could allow streaming together different steps,^14^ as well as integration into a biological safety cabinet to enable sterile, long-term workflows.

Currently, OSCAR’s protocols must be written in C++, which can be difficult for users without programming experience. To facilitate protocol creation by nonexperts, we plan to encapsulate these C++ actions within within the BehaviorTree.CPP framework,^15^ which uses an easy-to-edit XML format instead of source code. Migration to this framework would also provide the *Groot2* ^16^ visual editor, allowing users to build and modify protocols by arranging blocks on a graphical interface, thus lowering the barrier to entry while keeping the flexibility of the existing system. Although LLMs could, in principle, be added on top of the current interface to translate natural-language instructions into protocol steps and already lower the barrier to entry, we chose to use the DSL as the primary protocol format to ensure determinism, traceability, and reproducibility. Our main concern was that LLM-generated procedures are not inherently deterministic and may vary in decomposition or ordering of actions, introduce unintended steps via hallucinations,^17^ or even fail outright,^18^ which complicates verification and validation in experimental workflows. Nonetheless, integrating LLMs with a structured and verifiable DSL^19^ remains an interesting direction for future work.

Calibration is required for operations that demand high Cartesian accuracy, such as attaching a pipet tip onto the shaft and performing colony picking. In our experience, achieving robust calibration through trial-and-error refinement can take a few hours. For inexperienced users, this process can be particularly time-consuming and may require substantial trial-and-error before converging to an acceptable calibration. Additional details and practical guidance to ease this process are provided in the Supporting Information.

Along with the incremental software and hardware improvements, a future goal is to introduce tool-changing capabilities for end effectors, for example, to swap between a single and a multichannel pipet during a protocol. We also designed our platform for easy scalability, as many manipulations will require either more throughput than one robotic arm can achieve or more working space than a single arm can reach. The MoveIt Task Constructor software used for motion planning also enables collaboration between multiple robotic arms to perform more complex tasks.

The success of open-source ecosystems in diverse fields, ranging from Wikipedia’s democratization of knowledge to the widespread adoption of ROS in robotics, demonstrates the power of community-driven development.^6,20,21^ These collaborative initiatives can not only outperform proprietary counterparts but have also fostered accessibility, rapid innovation, and interoperability. By drawing inspiration from successful open-source models, the scientific community has the opportunity to develop a robust, adaptable, and user-friendly platform for laboratory automation. Moving forward, fostering an open, community-driven approach in lab robotics will require concerted efforts in standardization, modular design, and shared infrastructure. If successful, this paradigm shift could redefine the development and deployment of laboratory automation, ultimately benefiting the broader scientific community.

## Material and Methods

### Robot Hardware

The OSCAR platform was assembled using a UR3 robotic arm from Universal Robots (academic price CAD 25,000 ≈ USD 18,750). It is mounted on an optical breadboard plate, model SA2–18 × 30 from Newport (USD 670), serving as the stable base and anchoring surface. Attached to the end of the robotic arm is a dual adapter (model AGC-APL-159–002, Robotiq; CAD 300 ≈ USD 225), supporting:

1. A gripper, model Robotiq 2F-140 (CAD 3,731 ≈ USD 2,799),
2. A torque-force sensor, model Robotiq FT300 (CAD 6,000 ≈ USD 4,500),
3. An Intel RealSense D415 3D camera (CAD 427 ≈ USD 300).

On the opposite side of the adapter, a custom-built electronic pipet is mounted, designed for precise automated liquid handling. Detailed printing and assembly plans are available here:

1. https://github.com/rodrigue-laboratory/pipette-tool-cad
2. https://github.com/rodrigue-laboratory/pipette-tool-pcb

The electronic micropipette, depicted in Figure 1B, was built using the mechanism of a Gilson PIPETMAN 20 to 200 μL manual micropipette (CAD 700 ≈ USD 510). Piston displacement is controlled by a micromotor, actuated by an Arduino MKR0, and enclosed within a 3D-printed frame. All tips and media used were previously sterilized by autoclave.

### Robot Control

The robotic system described in this study was implemented on Ubuntu 20.04 and ROS Noetic. Users should begin with the OSCAR package, which serves as the central launch hub for the OSCAR platform. Robot motion planning and execution relied primarily on the ROS packages MoveIt, MTC, and cartesian_controllers. Control and communication with the Universal Robots robotic arm were handled by the Universal_Robots_ROS_Driver. Vision capabilities were provided by the realsense-ros package, which integrates data from Intel RealSense cameras, while manipulation tasks involving gripping were facilitated by the robotic package. Additionally, data processing, numerical computations, and image analyses were performed using SciPy and OpenCV libraries. Table 1 shows a complete list of the required packages.

**Table 1:** 

Package	Description	Link
oscar	Central launch hub for the OSCAR platform	repo
cartesian_controllers	Cartesian motion/impedance controllers	repo@c1526e5
control_msgs	ROS control message/action definitions	repo@c776cc6
deterministic_trac_ik	Deterministic TRAC-IK kinematics plugin	repo@f80c3d4
oscar_description	URDF and meshes for the OSCAR platform	repo
oscar_robot_setup	Bringup/launch/config for lab setups	repo
oscar_task	Build and execute the three biology protocols	repo
oscar_ur_launch	UR-specific launch files and configs	repo
oscar_vision	Vision nodes/pipelines (e.g., RealSense)	repo
moveit	MoveIt (motion planning framework)	repo@ba67fc3
moveit_task_constructor	MTC – MoveIt Task Constructor (task planning)	repo@0b00477
pipet-tool-cad	Pipette CAD drawings	repo
pipet-tool-pcb	Pipette PCB drawings	repo
pipet-tool-sw	Pipette firmware/driver	repo
realsense-ros	Intel RealSense ROS wrapper	repo@b14ce43
robotiq	Robotiq gripper/FT drivers and msgs	repo@0b2f924
ros_colony_morphology	Colony detection and morphology analysis	repo@7cf4f3c
ros_pipette_tool	Pipette tool driver/firmware interface	repo@0ef0586
sodf	SODF – Semantic Object Description Format	repo@e90e050
task_space_feedback	Task-space feedback control utilities	repo@d2f86c0
Universal_Robots_ROS_Driver	Official UR ROS driver	repo@d73f7f7
vision_opencv	OpenCV ROS wrapper	repo@cfabf72

### Biological Materials

The strain used for transformation of the plasmid is *E. coli* MM294, prepared according to this protocol: https://mcmanuslab.ucsf.edu/protocol/rubidium-chloride-competent-cell-protocol. LB Agar plates were prepared using commercially available powder (Biobasic Cat.#: SD7003) as per the manufacturer’s instructions. Plates were supplemented with 34 μg/mL of chloramphenicol to select plasmid transformation.

The primers used for PCR amplification were oAC1034/1035 (Fragment 1, 1999 bp) and oAC1036/1037 (Fragment 2, 2103 bp). For assembly verification, primers oAC1035/1041 (3064bp) were used. The PCR mix used is a homemade preparation of the Neq2 × 7 open-source fusion polymerase, prepared as instructed in the original publication.^16^

**oAC1034** GCATTAAGCGCGGCGGGTG

**oAC1035** CGGGCTCATGATCCTAGAAATATTTTATC

**oAC1036** CTTATTAATCAGATAAAATATTTCTAGGATCATGAGCCCGttacgccccgccctgccac

**oAC1037** CACGCTGCGCGTAACCACCACACCCGCCGCGCTTAATGCgCGGCCGCttattcaacatag

**oAC1041** TCGGTCAGTTTCACCTGATTTACGTAAAAACCacctaggTCGGT

For DNA assembly the NEBuilder HiFi DNA Assembly Master 2x Mix was used as recommended by the manufacturer.

## Supplementary Material





## Notes

Systematic error of pipetting across commercial pipette ranges and a unified custom pipet (Figure S1); solving a picking task using the MTC library (Figure S2); dual-mode planning architecture (Figure S3); motion-phase template for robotic manipulation, illustrated via tip insertion (Figure S4); colony location process from a 2D image (Figure S5); main quantitative morphology metrics extracted from individual colonies (Figure S6) (PDF)
Protocols implemented on the OSCAR platform written in a human readable format (PDF)
