# Supplementary material for: OSCAR: A Modular Open-Source Robotic Platform for Biological Laboratories
Source: ACS Synth Biol. 2026 Mar 9;15(3):1062–72. doi: 10.1021/acssynbio.5c00733 (PMC13010791; doi:10.1021/acssynbio.5c00733)
Supplement: Supplementary file 1 [file sb5c00733_si_001.pdf]

Supplementary Material for

OSCAR: A Modular Open-Source Robotic Platform for Biological Laboratories

David Pivin[1], Antoine Champie[2], Mirco Plante[3], François Ferland[1], François Michaud[1], Sébastien Rodrigue[2*]

Supplementary figures:

- **Supplementary Figure S1:** Systematic error of pipetting across commercial pipette ranges and a unified custom pipette.
- **Supplementary Figure S2:** Solving a picking task using the MTC library.
  Supplementary Figure S3: Dual-mode planning architecture.
- **Supplementary Figure S4:** Motion-phase template for robotic manipulation, illustrated via tip insertion.
- **Supplementary Figure S5:** Colony location process from a 2D image.
- **Supplementary Figure S6:** Main quantitative morphology metrics extracted from individual colonies.

Supplementary discussion:

- Accuracy in computed tasks versus manually-teached ones.
- Calibration process.
- Motion planning and control architecture
- Semantic Object Description Format (SODF)
- Task coordination
- Task execution
- Dual mode motion planning

Videos showcasing the platform in action:

- Overview of the platform: https://youtu.be/tnEEWbkXtyo
- PCR Amplification and Validation: https://youtu.be/0sEpT15sNio
- Gibson Assembly and Bacterial Transformation: https://youtu.be/zaVK677Wxpc
- Colony Picking and Construct Validation: https://youtu.be/W8uVG6fVSUM

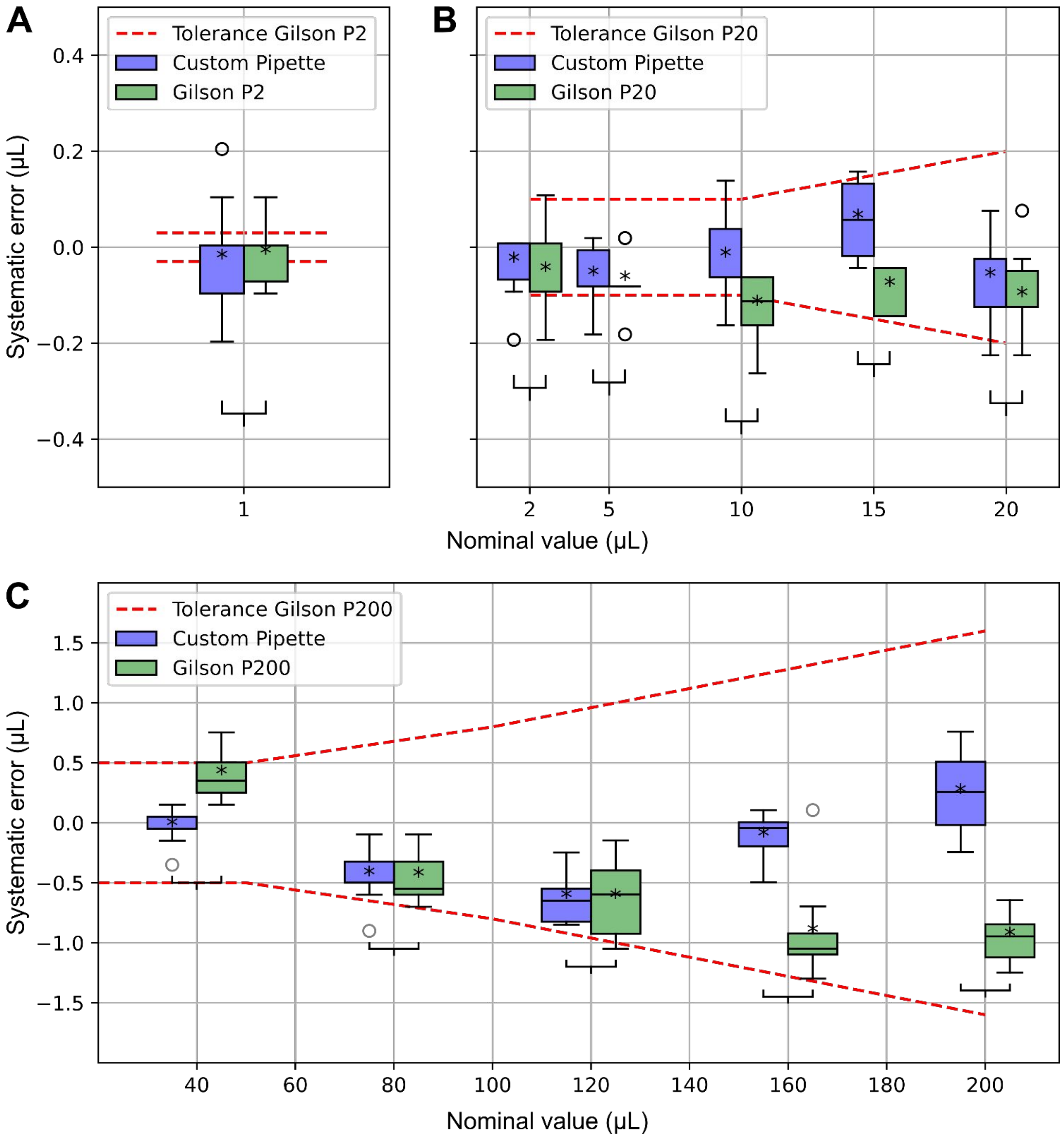

Figure S1: Systematic error of pipetting across commercial pipette ranges and a unified custom pipette. Boxplots show the systematic error (bias) in dispensed volume for a single custom robotic pipette (blue) compared to three commercial Gilson pipettes (green), each dedicated to a specific volume range: (**A**) P2 (1 µL), (**B**) P20 (2–20 µL), and (**C**) P200 (20–200 µL). The custom pipette was operated across the full range without switching hardware, while the commercial system required three separate pipette models. Red dashed lines indicate the maximum allowable systematic error based on Gilson specifications for each nominal volume. Black asterisks mark the sample mean with box and whiskers representing quartiles. Each target volume was assessed using ten independent replicates. The custom pipette consistently met or exceeded commercial accuracy across all tested volumes, staying within tolerance limits while covering the full dynamic range with a single actuator. All volume measurements were corrected using the Z-factor to account for air buoyancy effects during gravimetric calibration, in accordance with ISO 8655. Note: For measurements below 20 µL (panels A and B), the precision of the balance used did not fully meet ISO 8655 recommendations, so these results should be interpreted with caution.

A) Task description

```
pick task
  ↕ current state (A)
  ↓ open hand (B)
  ⊖ move to pick (C)
  ↕ pick object
      ↑ approach object (D)
      ↕ grasp pose IK (E)
          ↕ generate grasp pose (F)
      ↓ cloe hand (G)
      ↓ lift object (H)
```

↑ forward propagating stage
↓ backward propagating stage
⊖ connecting stage

B) Planning

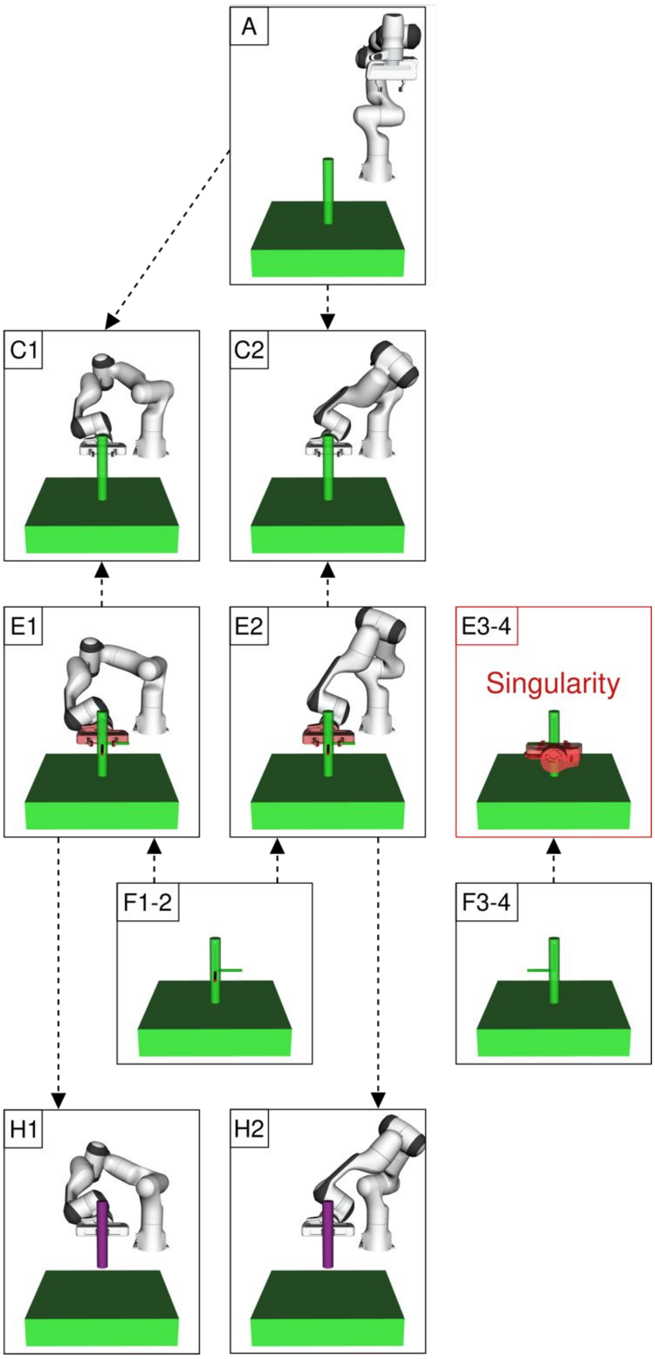

C) Solutions

	Stage cost			
Stage	Path 1	Path 2	Path 3	Path 4
A	0	0	0	0
B	0	0	0	0
C	6.0	8.0	-	-
D	1.3	1.5	-	-
E	7.2	10.1	inf	inf
F	0	0	0	0
G	0.03	0.03	-	-
H	0.6	0.6	-	-
Total	10.1	19.4	-	-

Figure S2: Solving a picking task using the MTC library. A) Task description and propagation stages for a robotic pick task. The propagation direction of planning states is indicated by arrows. The connection stage will try connecting the B nodes with the D nodes. Hierarchy is indicated by indentation. **B**) Multi-path planning graph generated using MTC, showing possible transitions between motion stages. The process starts with nodes A and F. Node A forwards its solution to the B nodes, while the F nodes generate solutions that are passed backward to earlier stages (E1-4). The propagation continues until a stop criterion is met: a timeout or a maximum number of solutions. Node B1-4 has no cost because the gripper is already open to the desired position. Node E3–4 (red) illustrates a failed solution due to a kinematic singularity, thus halting its forward and backward propagation. Nodes B1-4, D1-2, and G1-2 are not shown for better clarity. **C**) Stage-wise cost (shortest path) for candidate solution paths. Path 1 (green overlay) achieves the lowest overall cost (10.1), while Path 2 yields a valid but suboptimal solution. Paths 3 and 4 are invalid due to infeasible inverse kinematics.

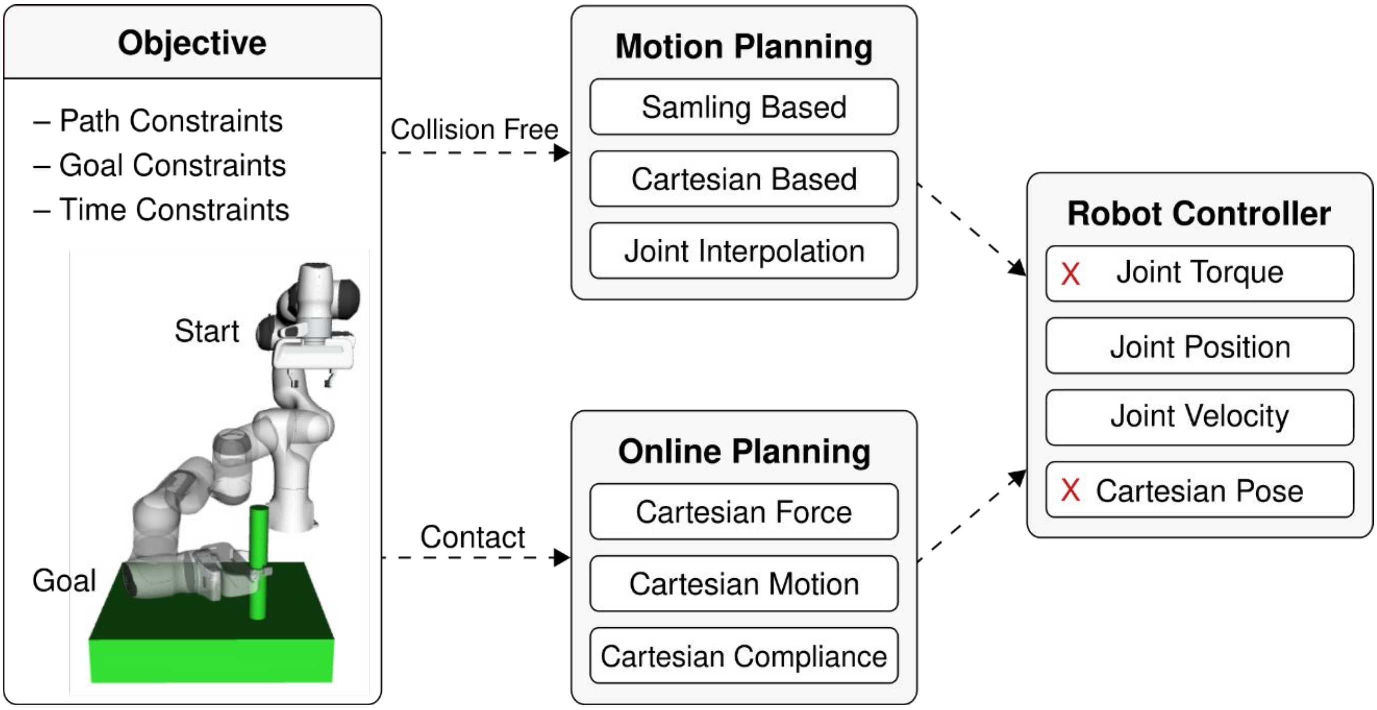

Figure S3: Dual-mode planning architecture. The system integrates both offline and online planning strategies to meet task objectives such as collision avoidance, path, time and goal constraints. Offline motion planning includes sampling-based, cartesian-based, and joint interpolation methods to generate precomputed paths. These are fed into the trajectory planner (not shown in figure), which produces joint-space commands for the robot controller (e.g., joint position and/or velocity). For force-sensitive interactions, the system transitions to online planning modes using real-time cartesian motion, force, or compliance control. These modes directly interface with the robot controller to achieve stable and responsive behaviour during contact using the joint position or the joint velocity interfaces. To maintain compatibility across different hardware platforms, we deliberately avoid using vendor-specific robot cartesian controllers (red X mark, Cartesian Pose module). Furthermore, joint torque control is often unavailable or disabled on standard industrial robots (red X mark, Joint Torque module).

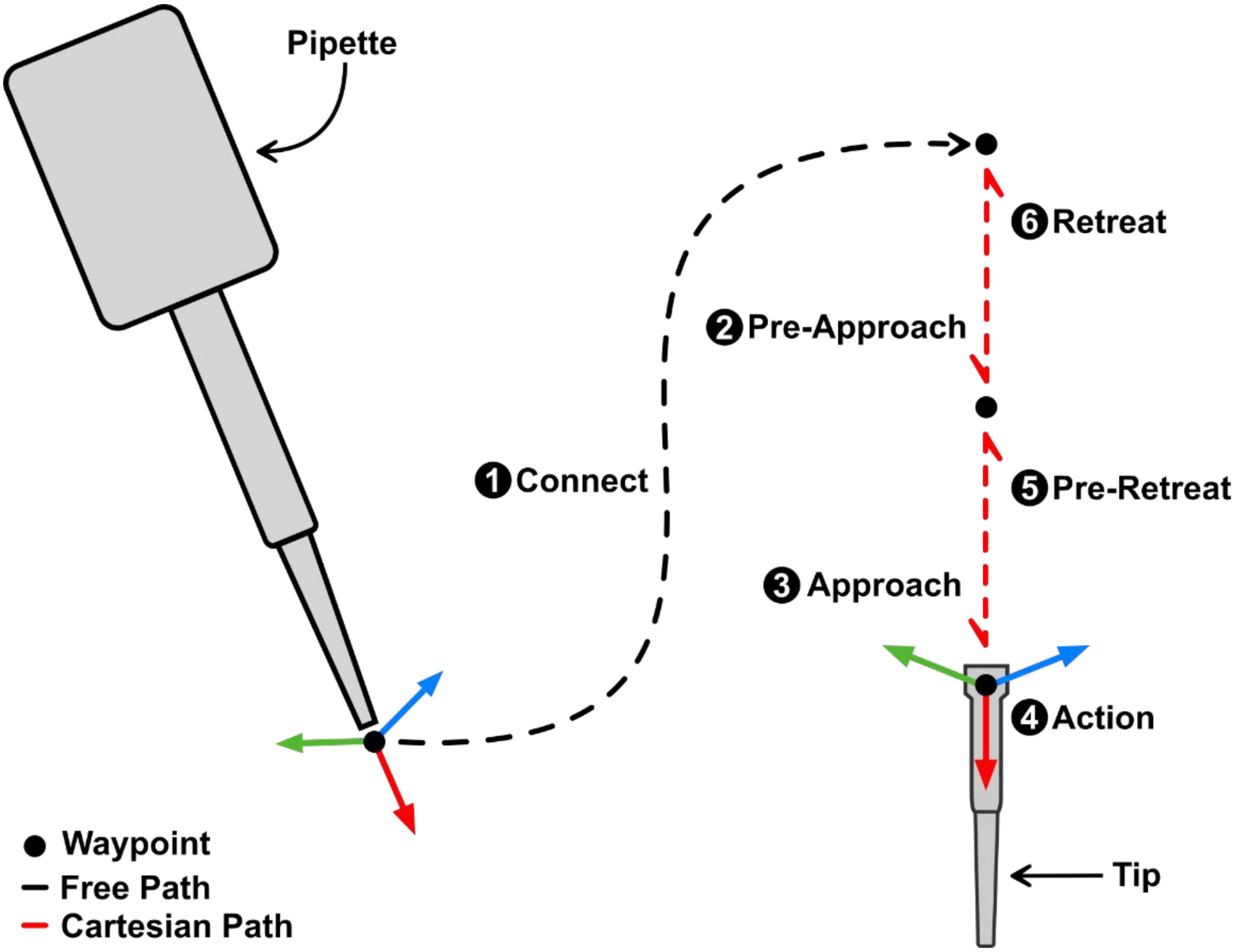

Figure S4: Motion-phase template for robotic manipulation, **illustrated via tip insertion**. Step 1) Connect: a free motion plan from the current state to the pre-approach start position; Step 2) Pre-Approach: a Cartesian motion plan to a desired distance; Step 3) Approach: a Cartesian motion plan to the target position; Step 4) Action: the execution of the desired operation; Step 5) Pre-Retreat: a Cartesian motion plan upwards by a specified distance; and Step 6) Retreat: a final Cartesian motion plan upwards to the end position. This modular decomposition facilitates efficient collision detection and avoidance, particularly in the critical phases between Step 3 and Step 4, where contact may be required. Without this structure, collision detection could prematurely halt planning due to the detection of an illegal contact. Additionally, motion in Step 3 and Step 4 can be substituted with a real-time Cartesian planner to achieve a target force or compliance, which is beneficial for tasks requiring precise control over interaction forces. To account for slight deviations introduced by online control, the robot then transitions to the start of Step 6 using a joint-space interpolation. Note: motion in Step 3 and Step 5 is usually much smaller than Step 2 and Step 6.

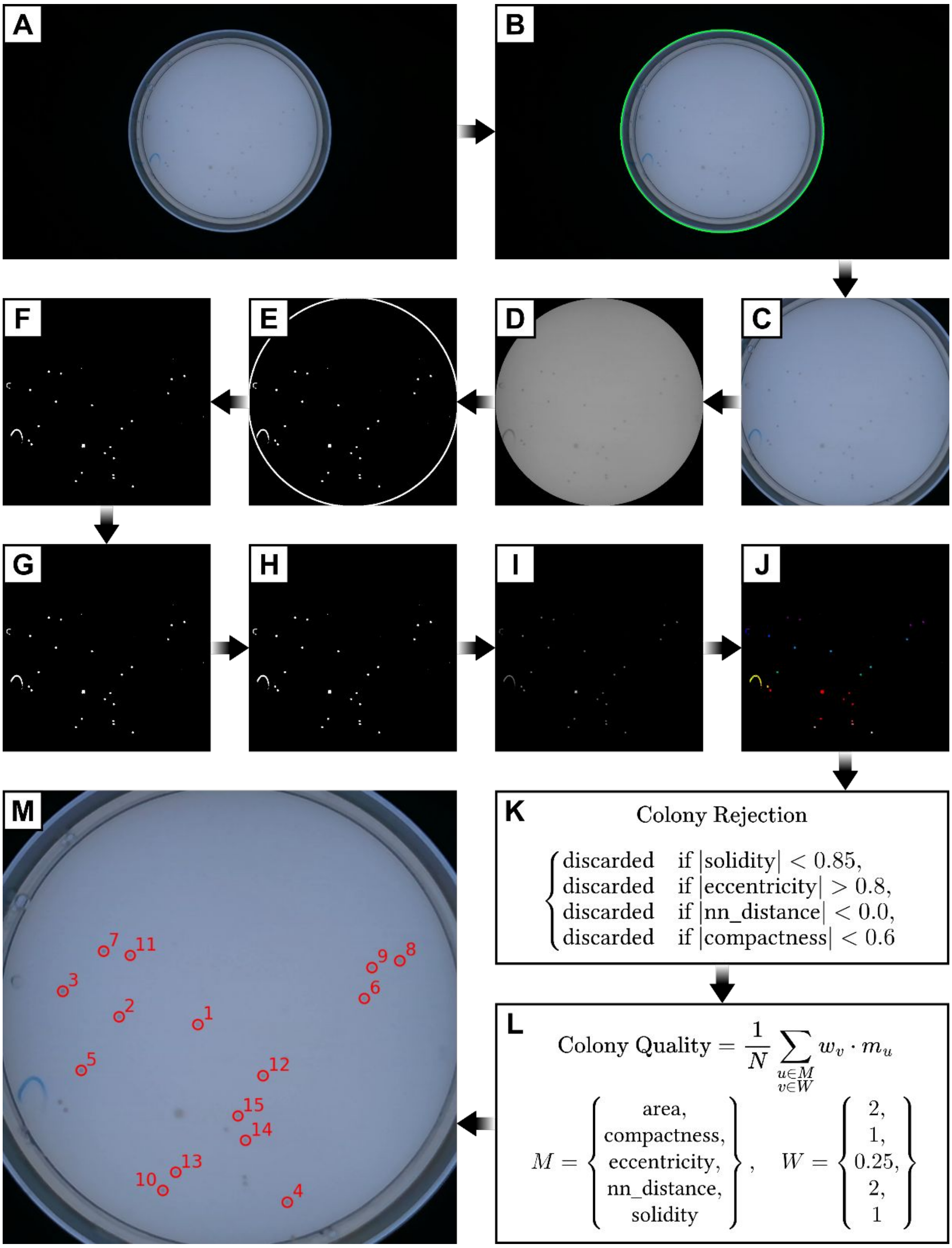

Figure S5: Colony localization from a 2D image. A) Input RGB image. The image is first converted to grayscale and rescaled to reduce computational cost. **B)** Petri dish boundary detection using the Hough Circle Transform, after Canny edge detection. The circular boundary is refined by sampling equidistant points along the perimeter and optimizing them based on local intensity contrasts. **C)** Cropped image, restricted to the region of interest within the refined Petri dish boundary. **D)** Masking of regions outside the dish to exclude irrelevant background. **E)** Adaptive binary thresholding to segment potential colony regions. **F)** Filtering step to remove contour artifacts introduced during binarization. **G)** Morphological opening (erosion followed by dilation) is applied to eliminate small noise particles. **H)** Morphological closing (dilation followed by erosion) fills small holes within segmented objects. **I)** Euclidean distance transform, applied to the binary mask, computes the distance from each pixel to the nearest foreground object. **J)** Watershed segmentation is seeded using local maxima from the distance map, followed by computation of region-specific morphology metrics. **K)** Segmented objects are filtered based on user-defined thresholds applied to computed metrics. **L)** A custom colony quality score is computed by combining features such as area, compactness, eccentricity, solidity, and inter-colony proximity. **M)** The top-ranked colonies are selected for picking.

Label	Colony Quality	Compactness	Collision Distance	Area	Solidity	Eccentricity	Centroid (x / y)
**1**	0.85	1.09	118.74	45	0.92	0.43	373.8 / 311.2
**2**	0.78	1.06	90.91	46	0.88	0.33	361.3 / 185.5
**3**	0.74	1.18	66.40	51	0.98	0.26	320.5 / 95.6
**4**	0.72	1.06	65.71	46	0.92	0.29	657.8 / 454.0
**5**	0.71	1.21	53.28	45	1.00	0.00	447.0 / 125.0
**6**	0.64	1.13	43.38	42	0.95	0.47	332.1 / 577.0
**7**	0.63	1.12	35.70	46	0.94	0.36	256.5 / 160.7
**8**	0.62	1.09	38.08	47	0.92	0.61	271.8 / 633.6
**9**	0.62	0.97	38.08	45	0.90	0.50	282.7 / 589.2
**10**	0.00	1.83	68.09	8	1.00	0.61	244.9 / 274.1
**11**	0.00	1.77	-2.39	9	1.00	0.99	296.8 / 22.6
**12**	0.00	1.61	-4.02	46	1.00	0.92	298.3 / 23.9

Table S6: Main quantitative morphology metrics extracted from individual colonies. Labels 1 through 9 correspond to ranked isolated colonies that do not contact adjacent structures or neighboring colonies. The computed metrics include compactness, collision distance, projected area, solidity, eccentricity, and centroid position in picture coordinates. Colonies 10, 11 and 12 are examples of colonies excluded based on filtering criteria: colonies 10 and 11 were rejected based on abnormal size while 11 and 12 were rejected due to a negative collision distance, indicating physical overlap with a neighboring colony. Valid colonies are shown in Supplementary Figure S6M, whereas rejected colonies are not displayed.

Supplementary Discussion

Accuracy in computed tasks versus manually taught ones

The UR robot is equipped with a teach pendant, which serves as the programming interface for controlling its operations. This configuration provides a straightforward approach to planning deterministic tasks without specialized robotics expertise. The planning process is characterized by its simplicity, determinism, and computational efficiency, as it does not incorporate collision detection mechanisms. Additionally, there is no need for environmental characterization, such as defining the table and labware. The system delivers optimal absolute positional accuracy, corresponding to the robot's repeatability (±0.1 mm). However, a key limitation of this approach is the need for manual operation to record waypoints for each interaction. While this process is suitable for repetitive protocols, it becomes problematic when modifications occur, such as relocating a tip rack, requiring the resaving of affected waypoints. This method is also ill-suited for tasks involving unknown waypoints, such as colony picking. Furthermore, precise operations will need to be retrained if the robot is replaced with another of the same model, as all waypoints must be rescanned for a different unit.

In the OSCAR platform, tasks are fully computed rather than being manually taught. This approach requires calibration of the robot cell, particularly when operations demand high Cartesian positioning precision. For instance, attachment of a tip onto the pipette shaft requires a precision of ±0.5 mm, and colony picking, which involves a 1 mm diameter target. Since the UR3 robot offers a repeatability of ±0.1 mm, one might assume that these precise operations would pose no challenge. This assumption holds true in traditional teaching-based methods, where tasks are programmed by jogging the robot or manually guiding it. However, given that our task goals are computationally determined, absolute accuracy is required. It is critical to establish the exact location of a station (e.g., table) relative to the robot base, as well as the position of tools (e.g., pipette tip, gripper, camera) relative to the robot's mechanical flange. While various commercial solutions for robot calibration using metrology devices exist, even the most economical options cost tens of thousands of dollars.

Calibration process

The complete system setup requires four distinct calibration procedures, which a new user with basic robotic skills can perform in approximately 12–15 hours, depending on the number of iterations required. In our system, we calibrated the table mounting holes relative to the robot base using a 3D touch probe mounted on the robot's end effector. For each target hole, the operator jogged the robot until the probe contacted the outer diameter of a precision single-end stud inserted into the corresponding threaded location, and the resulting robot pose was recorded. To improve robustness, we recommend collecting at least four contact samples per hole from distinct approach directions. In our setup, calibrating 16 holes required approximately 2–3 hours, including estimating hole locations and updating the table semantic description. The tool center point (TCP) calibration of the pipette tip and gripper (in the closed state) was performed using the UR dashboard program. The operator repeatedly brought the tool into contact with a fixed, precisely defined reference feature on the table (e.g., a table corner) using four distinct tool orientations, and iteratively refined the TCP estimate until consistent coincidence was achieved across orientations. As this is a manual, iterative process, it can take up to 2 hours, depending on the number of trials needed. For extrinsic camera calibration, we utilized the hand-to-eye method from the MoveIt Calibration package. These free calibration procedures are somewhat susceptible to user error, such as poor-quality targets, insufficient pose space coverage, or inadequate sampling. This is the most complex calibration procedure: it needs at least 8 waypoint samples to obtain a well-conditioned estimate. In practice, the procedure may require up to approximately four hours, including multiple trial-and-error iterations, until the calibration converges to a low residual error. The table-to-robot base calibration achieved an accuracy of approximately ±0.07 mm. The TCP calibration reached a precision of ±0.4 mm and the camera reprojection error was ±1 mm. While the pipette tip insertion process (**Fig. 3A**) functions effectively within the required precision limits, aided by the flexibility of the pipette shaft and the semi-rigid tip, the colony picking operation fails due to accumulated precision errors in the camera

and TCP calibration (±1.4 mm). To mitigate this, a repicking strategy around the initial target was employed. Finally, the relationship between pipetted volume and linear displacement was calibrated across three volume ranges (1–2 µL, 2–20 µL, and 20–200 µL). The calibration and validation took roughly 4 to 6 hours. The complete methodology is described in the GitHub repository README (https://github.com/rodrigue-laboratory/pipette-tool-sw), which also documents a command-line interface (CLI) that facilitates setup, calibration, and validation. The framework supports the definition of an arbitrary number of user-defined calibration ranges.

Motion planning and control architecture

Motion generation and execution are built on ROS ecosystem, leveraging MoveIt for kinematics, collision checking, and motion planning, together with MoveIt Task Constructor (MTC) to structure complex manipulation actions as modular, sequential task graphs. Protocols are written using a Domain-Specific Language (DSL) that encodes workflows as sequences of well-defined, object-anchored “behavior blocks” expressed in domain-relevant verbs (e.g., open lid, grasp, insert tip, aspirate, dispense/mix, spread, eject tip), with explicit parameters for targets, volumes, speeds, and force settings. Protocol logic is expressed through a library of C++ nodes, each implemented as a self-contained, strongly typed unit encapsulating its inputs and outputs. Users can readily reuse existing nodes or compose more complex ones. Each node follows the behavior tree paradigm, enabling structured composition through well-defined control flow and interfaces.

Semantic Object Description Format (SODF)

To support a broad repertoire of contact-rich manipulation verbs, we developed the Semantic Object Description Format (SODF), a library for representing laboratory equipment through both geometric models and interaction affordances. In addition, SODF enables a scene graph by maintaining the relationships between child elements and their parent. For example, placing a Petri dish lid onto its corresponding Petri dish can be expressed by specifying two semantic frame identifiers—one associated with the lid and one with the dish—thereby implicitly defining the parent–child relationship during the insertion operation (e.g., `"petri_lid/insert/into"` and `"petri/insert/lid/from"`). SODF models are currently encoded in C++. While this approach ensures tight integration with planning and control, it remains less accessible to end users who wish to author or modify object descriptions. To address this limitation, we are migrating SODF toward an XML-based representation that allows users to more easily add, inspect, and edit object models without recompiling source code. In parallel, we are developing visualization tools based on RViz to enable interactive browsing of each object’s structural elements, reference frames, and semantic affordances. This visual exploration is intended to help users familiarize themselves with available object semantics and reuse them consistently when authoring new robotic protocols. Finally, tighter integration with the MoveIt planning scene and MTC remains future work and largely centers on extending the SODF database with a lightweight diff mechanism to support speculative planning, branching, and rollback.

Task coordination

Task coordination is handled by the MTC library, which represents robotic tasks as compositions of stage primitives grouped within containers. Stage primitives define the smallest planning or execution units (e.g., sampling a grasp pose, computing an approach motion, or validating a trajectory), while container type structure task logic. Serial containers execute stages sequentially and require complete end-to-end solutions, whereas parallel containers enable alternative or concurrent strategies, such as comparing planners, selecting fallback grasps, or executing independent actions simultaneously. Stage properties encode semantic information including target frames, planner and controller selection, and execution parameters. Target poses and interaction frames are derived from the SODF semantic object models. MTC is particularly well-suited for long-horizon protocols, as it exposes failures arising from specific action sequences early in the planning process while supporting solution branching, intermediate validation, and structured recovery strategies.

Task execution

Task execution is handled by a dedicated Task Manager, which aggregates all valid solution candidates generated by MTC and selects the solution with the lowest cumulative joint displacement, favoring efficient motions (**Fig. S2C**). Along with trajectory data, stage-specific execution parameters such as pipetting volume, aspiration and dispense speeds, and action type are passed directly to the corresponding low-level controllers. This allows motion commands and process-level operations to be executed through a unified interface. Because each MTC stage encodes its controller requirements as explicit properties, the Task Manager can automatically route each step to the appropriate controller (e.g., joint-space, Cartesian, or device-specific) without manual configuration. Additionally, MTC solutions represent planning-scene modifications as incremental scene-state deltas rather than full scene reconstructions. As a result, the planning-scene updates required for trajectory validation can be computed concurrently with controller execution. This concurrency reduces object-mesh processing as a computational bottleneck and enables seamless chaining of controllers without idle delays, while still ensuring a fully consistent planning scene at task completion.

Dual mode motion planning

Motion planning is performed using a dual-mode strategy **(Fig. S3)** that combines offline motion planning with reactive online control. Motion planning is primarily performed using the MoveIt library, which provides a suite of trajectory generators, inverse kinematics (IK) solvers, and collision checking mechanisms, which are then used by MTC. It is fundamentally a kinematic framework for non-contact behaviors. We selected deterministic planners such as the Cartesian Interpolator and Pilz Industrial Motion planners to generate rapid, predictable Cartesian trajectories and effectively address challenges such as cable routing. MoveIt also monitors the execution of the trajectory, stopping the controller if the robot deviates from specified tolerances. For contact interactions, such as aspirating from the bottom of wells or inserting a stylus into an enclosure, the system transitions to an online planning mode. In this mode, compliant motion is achieved through the `cartesian_controllers` ROS package[13], which provides delay-free, stable, and noise-tolerant force control. During these operations, collision checks are disabled and motion is constrained to small ranges to ensure safety. The same robot controller is used in both modes: it receives precomputed joint-space trajectories during non-contact motion, and dynamically generated trajectories based on real-time force sensing during contact.

Each protocol action, such as aspirating liquid from a well or grasping an object, is decomposed into a structured sequence of motion phases **(Fig. S4)**. The action is organized around successive free-space, Cartesian, and contact-aware motions, including an initial connection from the current state, a controlled pre-approach and approach toward the target, execution of the intended interaction, and staged withdrawal away from the workspace. These standardized steps enable robust and fast planning, facilitate collision avoidance near contact zones, and support real-time substitution with compliant control when interaction forces are required. This phase-based template introduces a trade-off between completeness and speed: by constraining each action to a small set of structured approach/engage/retract segments, we can rapidly screen Cartesian feasibility across many object-relative pose candidates, which is typically faster and more predictable than invoking randomized sampling-based planners (e.g., OMPL's RRT-family planners). This modular architecture makes the system ideal for robustly executing biological protocols by efficiently chaining together a wide variety of generic operations.
