# Supplementary material for: OSCAR: A Modular Open-Source Robotic Platform for Biological Laboratories
Source: ACS Synth Biol. 2026 Mar 9;15(3):1062–72. doi: 10.1021/acssynbio.5c00733 (PMC13010791; doi:10.1021/acssynbio.5c00733)
Supplement: Supplementary file 2 [file sb5c00733_si_002.pdf]

This document presents a plain text version of the protocols performed by the OSCAR platform.

- **Protocol #1:** Amplification of 2 fragments by PCR and verification on agarose gel.
- **Protocol #2:** Removal of template plasmid, Gibson assembly and transformation of the plasmids into competent E. coli, plating of the cells.
- **Protocol #3:** Colony picking and PCR verification of plasmid assembly.

Protocol #1: Amplification of 2 fragments by PCR and verification on agarose gel.

Setup

Plate Layout

- Gray wells represent positions currently inaccessible to the robot as they are obstructed by the lid.
- Names in the plate layout indicate the wells from which or to which the robot will pipette in the following protocols. Capitalized for clarity in text.
- If a volume is indicated in the plate layout, it represents an input for the protocol and has been manually pre-filled before the protocol starts.

	1	2	3	4	5	6	7	8	9	10	11	12
A												
B												
C	Template_1 (50 µl)	Primer_1_F (50 µl)	Primer_1_R (50 µl)									
D	Template_2 (50 µl)	Primer_2_F (50 µl)	Primer_2_R (50 µl)									
E												
F												
G	Reaction_1		Gel_1									
H	Reaction_2		Gel_2									

Tubes kept at 4°C

- 2X PCR mix (50 µl) :
  - H_2O : 16 µl
  - Buffer 5X Veraseq: 20 µl
  - dNTPs : 2 µl
  - Neq2X7 DNA Polymerase : 2 µl
- Sucrose Loading Dye (500 µl)
- 2 log DNA Ladder (500 µl)
- Water (500 µl)

Protocol

Prepare the PCR reactions

- Set the thermocycler to 4°C
- Transfer the 2x PCR mix into the plate
  - 12,5 µl from 2X_PCR_Mix to Reaction_1 **[G1]** (oAC1034/1035)
  - 12,5 µl from 2X_PCR_Mix to Reaction_2 **[H1]** (oAC1036/1037)
- Transfer water into the plate
  - 9,5 µl from Water to Reaction_1
  - 9,5 µl from Water to Reaction_2
- Prepare reaction 1
  - 1µl from Template_1 **[C1]** to Reaction_1
  - 1µl from Primer_1_F **[C2]** to Reaction_1

  - 1µl from Primer_1_R **[C3]** to Reaction_1
  - Mix with Up and down (10µl x 5 quickly)
- Prepare reaction 2
  - 1µl from Template_2 **[D1]** to Reaction_2
  - 1µl from Primer_2_F **[D2]** to Reaction_2
  - 1µl from Primer_2_R **[D3]** to Reaction_2
  - Mix with Up and down (10µl x 5 quickly)

Perform PCR reaction

- Start the PCR with the following program PCR_Neq2X7_2min
- Wait until PCR is done

Prepare the samples for the 1% agarose gel (Made manually with 80ml TAE, 5,3µl of SYBR green, 1/15000 dilution)

- Transfer 5µl of Loading Dye from Sucrose_Loading_Dye to Gel_1 **[G1]**
- Transfer 5µl of Loading Dye from Sucrose_Loading_Dye to Gel_2 **[G2]**
- Transfer 5µl of Amplified DNA from Reaction_1 to Gel_1
- Transfer 5µl of Amplified DNA from Reaction_2 to Gel_2
- Mix with Up and down (5µl x 5 quickly)

Transfer prepared samples into the agarose gel

- Transfer 5µl of Ladder from Ladder to Well #1 of the gel
- Transfer 10µl of DNA from Gel_1 to Well #2 of the gel
- Transfer 10µl of DNA from Gel_2 to Well #3 of the gel

Start the electrophoresis

- Set the power supply to:
  - 120 V
  - 40 min
- Start the power supply and wait 40 min.

Protocol #2: Removal of template plasmid, Gibson assembly and transformation of the plasmids into competent *E. coli*, plating of the cells.

Setup

Plate Layout

	1	2	3	4	5	6	7	8	9	10	11	12
A												
B												
C												
D	Reaction_1 (7µl)		Assembly									
E	Reaction_2 (4µl)											
F												
G	Heat-shock											
H												

Tubes kept at 4°C

- DpnI restriction enzyme (10µl)
- 2X Gibson mix (30µl)
- *E. coli* MM294 competent cells (~150µl)
- Water (500µl)

Tubes kept at room temperature

- LB 1,5ml

Petris dishes

- Transformation
- Negative control

Protocol

Gibson assembly

- Set the thermocycler to 4°C
- Transfer 6,5µl from Reaction_1 to Assembly **[D1]**
- Transfer 3,5µl from Reaction_2 to Assembly **[E1]**
- Transfer 11µl from 2X Gibson Mix to Assembly **[D3]**
- Transfer 1µl from DpnI to Assembly
- Mix with Up and down (6µl x 5 quickly)
- Set PCR at 37°C for 5 min (Lid = 105°C)
- Set PCR at 50°C for 60 min (Lid = 105°C)
    - During the assembly prepare the negative control:
        - Open control Petri Dish
        - Transfer 50 µl from the MM294 cells onto the control Petri Dish
        - Spread
        - Close control Petri Dish

Transform assembled DNA

- Transfer all volume from the Assembly well (~22µl) and transfer to the *E. coli* MM294 competent cells tube, mix with up and down (100µl x 5 slowly)
- Set PCR to 42C (Lid = Off)
- Transfer bacteria + DNA into the PCR machine (well labelled “Heat-shock” **[G1]**)
- Wait 1 min
- Transfer bacteria + DNA (“Heat-shock”) into the 1,5ml LB already prepared at room temperature
- Mix with rapid up and down pipetting (200µl x5 slowly)
- Close the thermocycler
- Turn off the thermocycler
- Wait 1h
- Open Petri Dish Transformation
- Mix with rapid up and down pipetting (200µl x5 quickly)
- Transfer 200µl from the LB tube onto the Petri Dish Transformation and spread the bacteria.
- Close the Petri Dish Transformation

Protocol #3: Colony picking and PCR verification of plasmid assembly.

Setup

Plate Layout

	1	2	3	4	5	6	7	8	9	10	11	12
A												
B												
C												
D												
E	Primer_F (50µl)		Master Mix		Water (100µl)							
F	Primer_R (50µl)											
G												
H	R_1	R_2	R_3	R_4	R_5							

Tubes kept at 4C

- 2X_Mix (100µl)
    1. H2O : 50 µl
    2. Buffer 5X Veraseq: 40 µl
    3. dNTPs : 5 µl
    4. Neq 2X7 DNA Polymerase : 5 µl
- Sucrose_Loading_Dye (500µl)
- Ladder (100µl)

Petri dishes

- Transformation

Protocol

Prepare the PCR master mix

- Set thermocycler to 4°C
- Transfer
    - 75 µl from 2X_Mix to Master Mix **[E3]**
    - 45µl from Water **[E5]** to Master Mix
    - 6µl from Primer_F **[E1]** to Master Mix
    - 6µl from Primer_R **[F1]** to Master Mix
    - Mix Master Mix with up and down (50µl x 5 quickly)
- Dispense the master mix
    - 24µl from Master Mix to R_1 / R_2 / R_3 / R_4 / R_5 **[H1>5]**

Colony picking

- Open the Petri Dish Transformation
- Take a picture and identify isolated colonies

- “Pick” one colony using a pipet tip and put it in R_1
    - Once the tip is inside the liquid resuspend the colony in the liquid by making up and downs (20µl x5 quickly).
- Pick 5 other colonies and put them in R_2 => R_5 following the same procedure.

Perform PCR reaction

- Start the PCR with the following program PCR_Neq2X7_2min
- Wait until PCR is done

Prepare the samples for the 1% agarose gel (Made manually with 80ml TAE, 5,3µl of SYBR green, 1/15000 dilution)

- Transfer 5µl of Loading Dye from Sucrose_Loading_Dye to R_1 => R_5, Mix with Up and down (10µl x 5 quickly), changing tip each time.

Transfer prepared samples into the agarose gel

- Transfer 5µl of Ladder from Ladder to Well #1 of the gel
- Transfer 10µl of DNA from R_1 => R_5 to Well #2 => Well #6 of the gel, changing tip each time.

Start the electrophoresis

- Set the power supply to
    - 120V
    - 40min
- Start the power supply and wait 40 min.
